# Supplementary material for: Tunable hybrid zeolites prepared by partial interconversion
Source: Nat Commun. 2023 Mar 6;14:1256. doi: 10.1038/s41467-023-36502-3 (PMC9988824; doi:10.1038/s41467-023-36502-3)
Supplement: Supplementary file 1 — Supplementary Information [file 41467_2023_36502_MOESM1_ESM.pdf]

Tunable hybrid zeolites prepared by partial interconversion

Monica J. Mendoza-Castro[1], Zhipeng Qie[2,3], Xiaolei Fan[2,4,5], Noemi Linares[1,*] and Javier García-Martínez[1,*]

[1] Laboratorio de Nanotecnología Molecular, Departamento de Química Inorgánica
Universidad de Alicante
Ctra. San Vicente-Alicante s/n, 03690 Alicante (Spain)

[2] Department of Chemical Engineering, School of Engineering
The University of Manchester
Oxford Road, Manchester M13 9PL (United Kingdom)

[3] Faculty of Environment and Life
Beijing University of Technology
Beijing 100124 (China)

[4] Nottingham Ningbo China Beacons of Excellence Research and Innovation Institute
University of Nottingham Ningbo China
211 Xingguang Road, Ningbo 315100 (China)

[5] Institute of Wenzhou
Zhejiang University
Wenzhou, 325006 (China)

Correspondence to: noemi.linares@ua.es; j.garcia@ua.es

TABLE OF CONTENTS:

1. Supplementary Experimental Methods 3

1.1. Catalytic evaluation 3

2. Supplementary Tables 4

2.1. Summary of all synthesized samples and their characterization 4

3. Supplementary Figures 5

3.1. Characterization of cetyltripropylammonium bromide 5

3.2. Ar physisorption at 77 K at low partial pressures (P/P_0) of samples prepared using CTPABr 5

3.3. TG analysis of samples prepared using CTPABr 6

3.4. Evaluation of the use of CTAB as SDA for the FAU to MFI transformation 7

3.5. Textural and structural characterization of samples prepared using TPABr 8

3.6. Textural and structural characterization of samples prepared using CTAB + TPABr 9

3.7. Evolution of the Si/Al ratio of the obtained materials 10

3.8. Textural and structural characterization of physical mixtures of FAU and MFI zeolites 10

3.9. Field Emission Scanning Electron Microscopy analysis of the samples 11

3.10. Transmission Electron Microscopy analysis of selected regions 11

3.11. Catalytic results of physical FAU:MFI mixtures and a mesoporous Al-MCM-41 material 12

3.12. ^{27}Al NMR analysis of selected materials 12

Supplementary References 13

1. Supplementary Experimental Methods:

1.1. Catalytic evaluation calibration. A schematic representation of the catalytic system used for the analysis is shown in Supplementary Figure 1a. The identification of the retention time (RT) of all products was performed by calibration using standard chemicals including benzene, toluene, *o,m,p*-xylene, 1,2,3- and 1,2,4-trimethylbenzene (TMB), 1,3- and 1,4-diisopropylbenzene (DiPBz) and 1,3,5-TiPBz (Supplementary Figure 1b**)**.

a

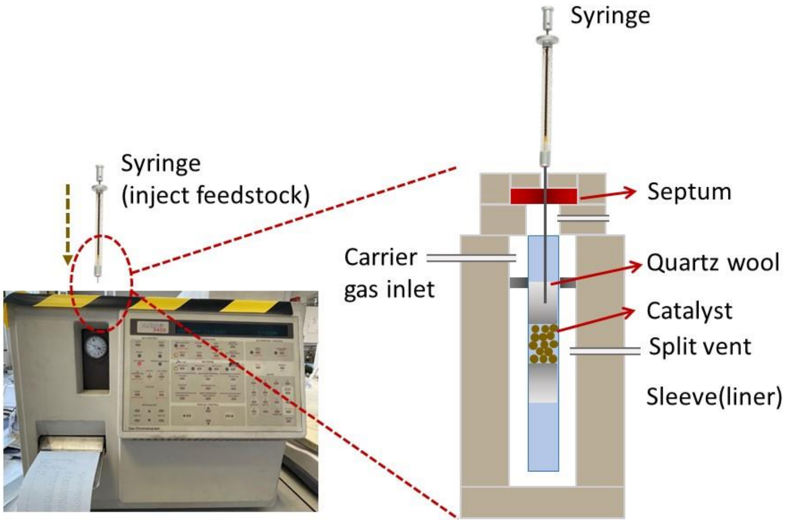

b

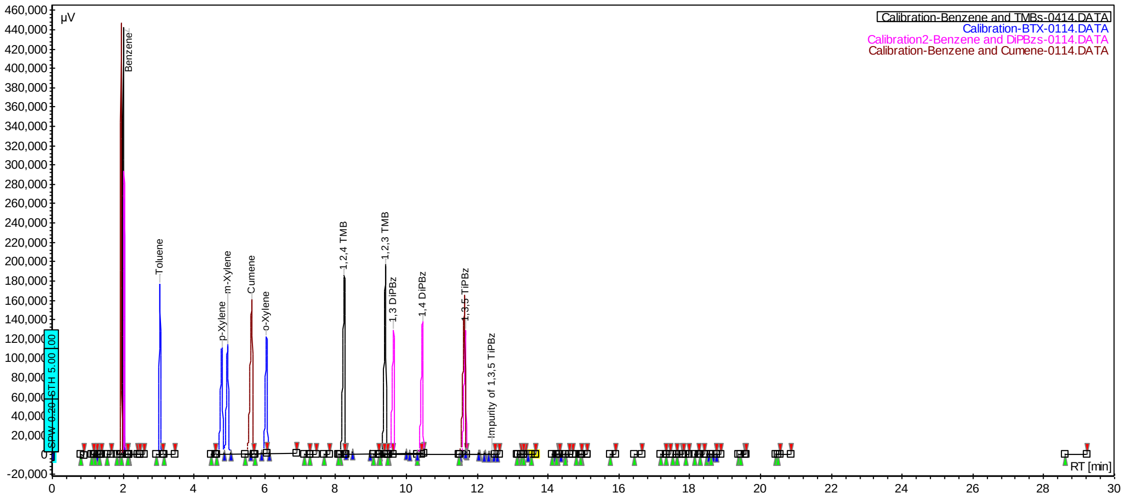

Supplementary Figure 1. (a) Schematic representation of the catalytic system used for the catalysts evaluation. (b) GC calibration chromatogram for the cracking of 1,3,5-triisopropylbenzene. Standards for benzene; toluene; o, m, p-xylene; cumene; 1,2,4-;1,2,3-; 1,3,5-TPB and 1,3-; 1,4-DPB were measured.

2. Supplementary Tables

2.1. Summary of all synthesized samples and their characterization. Samples were prepared by the four interzeolite transformation methods at different treatment times, and intermediate materials were thoroughly characterized. Supplementary Table 1 shows the details of all the samples involved in this study and their characterization.

Supplementary Table 1. Main properties of all the samples prepared using the different methods evaluated at different times.

Amine used	Treatment time (h)	Phase	%MFI[a]	Vmicro[b] (cm^3 g^{-1})	Vmeso[b] (cm^3 g^{-1})	Vtot[b] (cm^3 g^{-1})	Recovery yield (%)
–	CBV 780	FAU	0	0.25	0.24	0.49	–
TPABr	15	Am.	0	0.09	0.06	0.15	76.04
	18	Am. + MFI	44.5	0.12	0.06	0.18	90.15
	24	Am. + MFI	37.8	0.08	0.06	0.14	81.94
	48	MFI	73.4	0.12	0.05	0.17	88.47
CTPABr	39	Am.	0	0.02	0.38	0.4	85.48
	48	Am. + MFI	20.2	0.02	0.34	0.36	87.95
	72	Am. + MFI	49	0.05	0.26	0.31	79.02
	96	MFI	100	0.11	0.13	0.24	90.23
CTAB + TPABr	6	Am.	0	0.01	0.67	0.68	76.42
	12	Am. + MFI	35.8	0.01	0.5	0.51	84.11
	36	Am. + MFI	65.9	0.05	0.44	0.49	88.34
	168	Am. + MFI	70.8	0.09	0.4	0.49	82.51
CTAB	96	Am.	0	0	0.80	0.80	80.81
	240	Am.	0	-	-	-	84.27

a Calculated from the XRD analysis using a known amount of graphite as internal standard to normalize the spectra. The most crystalline MFI zeolite obtained after interzeolite transformation is completed, this is, zeolite HyZ-96, was defined as 100% MFI and used as reference to calculate the percentage of MFI in the other materials. **b** Cumulative pore volumes and pore-size distribution curves were calculated by using the DFT method (NL-DFT adsorption branch model). The total pore volume was obtained at the plateau of the cumulative adsorption pore volume plot at a relative pressure (P/P_0) of 0.9. Micropore volume was determined by NL-DFT as the volume adsorbed at pore sizes <2 nm, and the mesopore volume was calculated by subtracting the micropore volume from the total pore volume.

3. Supplementary Figures:

3.1. Characterization of cetyltripropylammonium bromide. The ^{1}H NMR (500 MHz) spectra were obtained in a Bruker Avance DRX300 by using $CDCl_3$ as solvent and TMS as the internal standard. The following abbreviations were used to describe peak patterns where appropriate: s = singlet, d = doublet, t = triplet q = quartet, sext = sextet, m = multiplet and br. = broad signal. ^{1}H NMR (300 MHz, $CDCl_3$) δ 3.46-3.15 (8H, br), 1.84-1.52 (6H, m) 1.4- 1.30 (2H, br), 1.30-1.18 (26H, br), 1.01-0.92 (9H, t) 0.84-0.75 (3H, t).

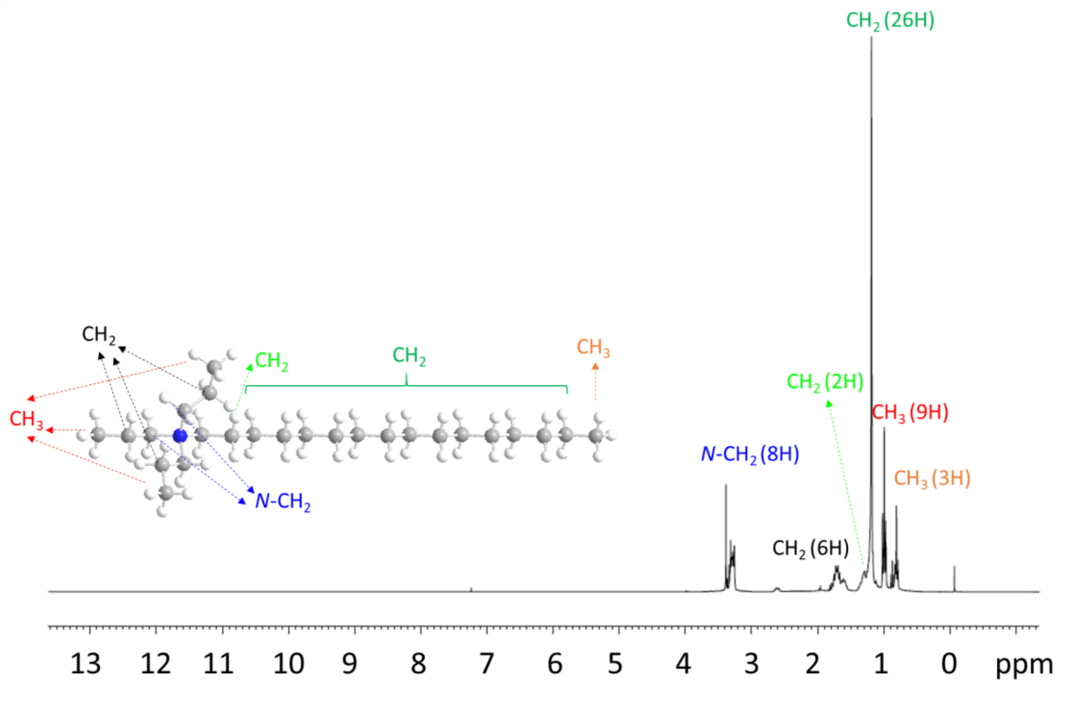

Supplementary Figure 2. ^{1}**H NMR spectra of the CTPABr in** $CDCl_3$ **and the corresponding assignation of the peaks.**

3.2. Ar physisorption at 77 K at low partial pressures (P/P_0) of samples prepared using CTPABr. Supplementary Figure 3 presents the adsorption profiles of the samples in the low P/P_0 range (10^{-7} – 0.01). The analysis of the adsorption in this region allows for studying the evolution of the size and shape of the micropore system in the samples.

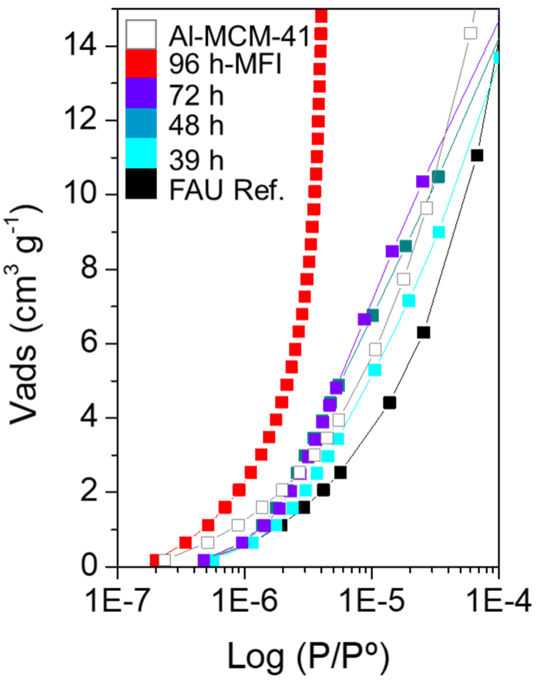

Supplementary Figure 3. Ar physisorption isotherms at -196 °C of samples prepared by treatment of a parent FAU zeolite with CTPABr at different times of treatment (shown in the legend).

3.3. TG analysis of samples prepared using CTPABr. Supplementary Figure 4 presents the TGA profiles of samples prepared at different time of crystallization. The analysis of the organic loss in the samples allows for studying the evolution of the amount of surfactant with the amount of MFI zeolite.

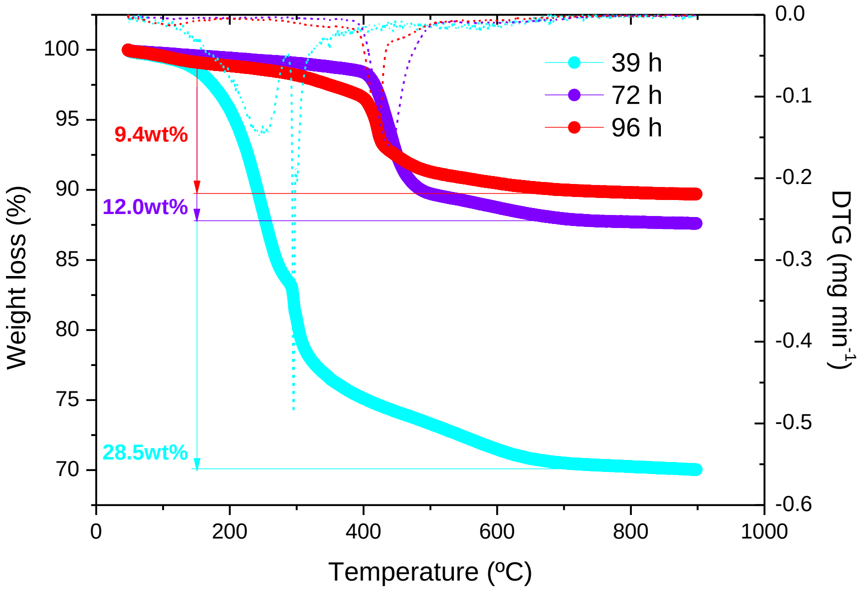

Supplementary Figure 4. TGA of samples prepared by treatment of a parent FAU zeolite with CTPABr at different times of treatment (shown in the legend).

3.4. Evaluation of the use of CTAB as SDA for the FAU to MFI transformation. As observed in Supplementary Figure 4, when only CTAB is used in the hydrothermal treatment of FAU no MFI zeolite was detected even at a very long time of treatment, indicating that the CTAB is not able to direct the synthesis of MFI under these reaction conditions.

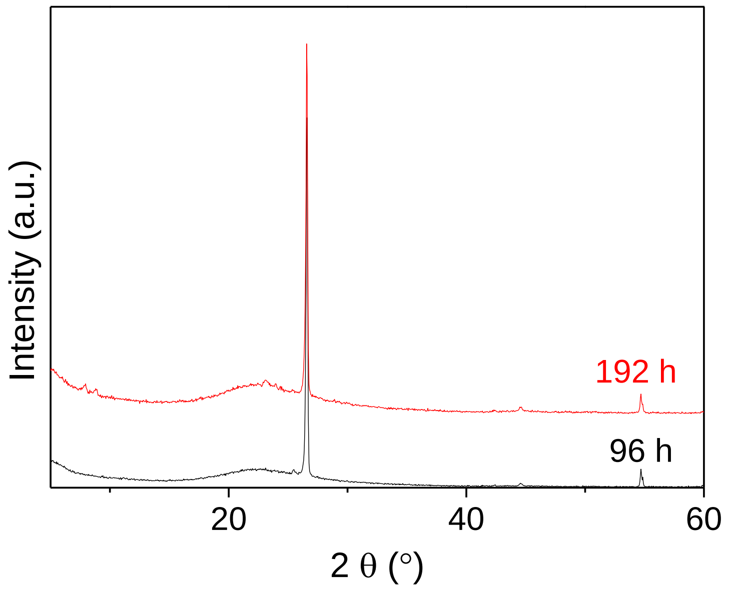

Supplementary Figure 5. XRD diffractograms of samples prepared by using CTAB as SDA during the FAU to MFI transformation at 4 and 8 days of hydrothermal treatment. Peak at 23 2θ (°) is due to the internal standard (graphite).

3.5. Textural and structural characterization of samples prepared using TPABr. Samples were prepared by interzeolite conversion using TPABr as SDA. As observed in Supplementary Figure 5, this FAU to MFI transformation does not evolve through mesoporous intermediates. In this case, the partially converted samples possess very low porosity.

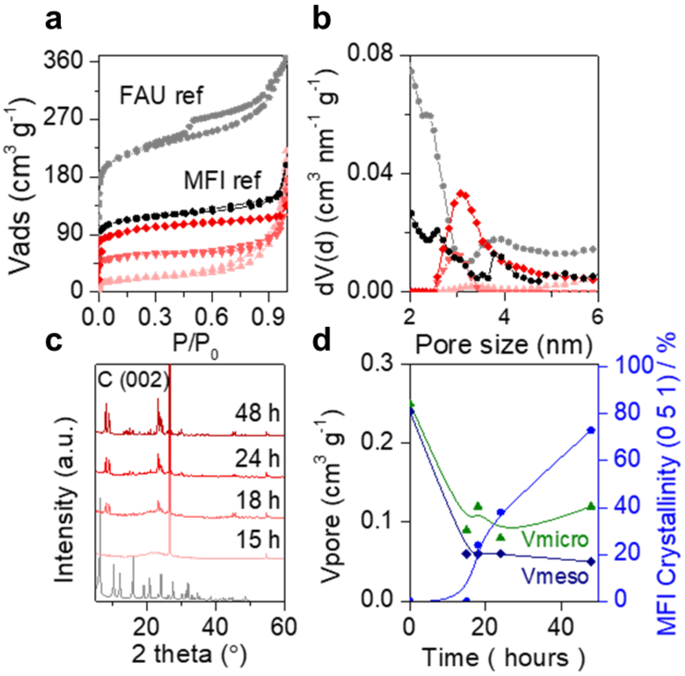

Supplementary Figure 6. Textural and structural characterization of samples prepared using TPABr. (a-b) Textural characterization of the samples prepared by this method: (a) N_2 physisorption isotherms at 77 K for samples prepared at different times (shown in the legend of (c)) in a linear scale; (b) the corresponding NL-DFT pore size distributions obtained from the adsorption branch of the isotherms. (c) XRD diffractograms obtained for the same materials, graphite, marked as C (002) in the diffractograms, has been used as an internal standard for the calculation of the %MFI crystallinity. (d) Evolution of the micropore (green triangles) and mesopore volumes (dark blue rhombus) and the %MFI crystallinity (blue circles) with the time of treatment.

3.6. Textural and structural characterization of samples prepared using CTAB + TPABr. Samples were prepared by interzeolite conversion using a mixture of CTAB and TPABr. In this case, the FAU to MFI transformation evolves through mesoporous intermediates with narrow pore size distributions, see Supplementary Figure 6. However, as can be observed in Supplementary Figure 6D, the evolution of the crystallinity stops at ca. 70% of MFI, and is not possible to reach a total transformation. Moreover, the TEM micrographs suggest the formation of two independent phases, an amorphous mesoporous one and a microporous MFI phase, Supplementary Figure 6e.

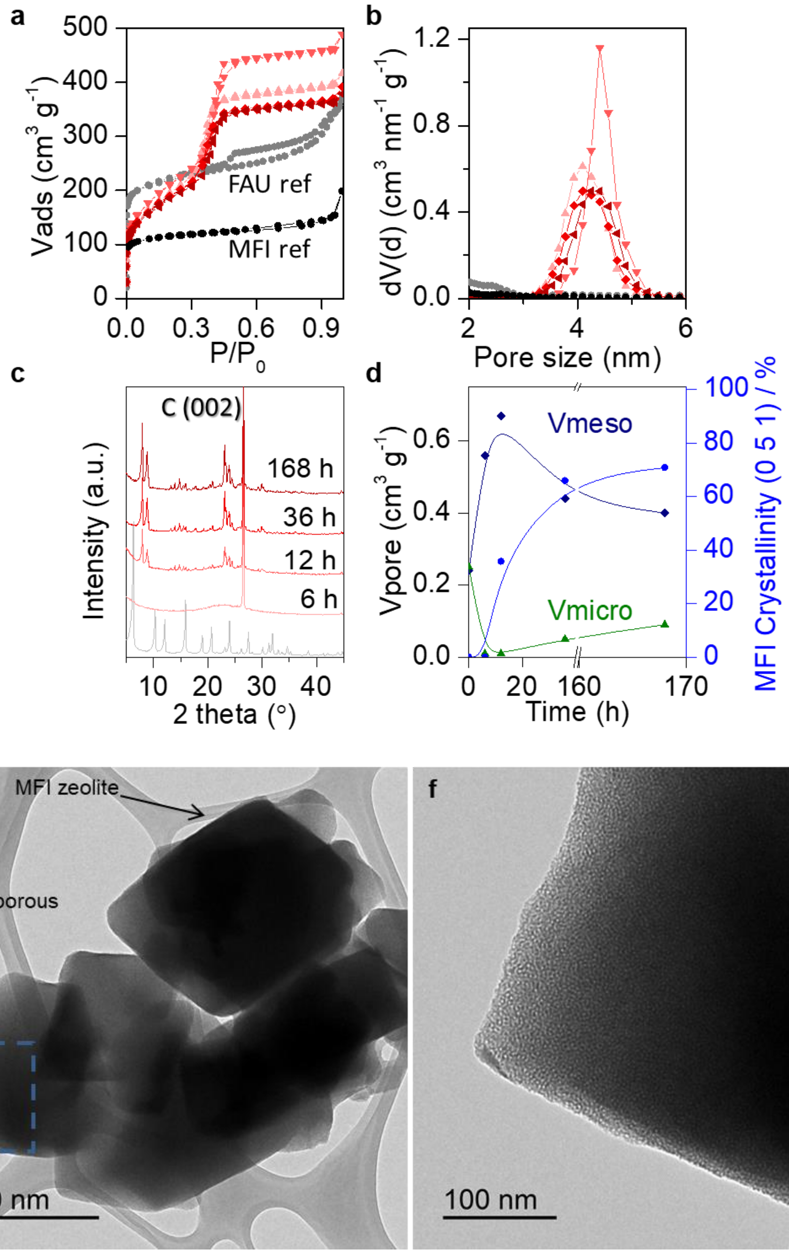

Supplementary Figure 7. Textural, structural and morphological characterization of samples prepared using CTAB + TPABr. (a-b) Textural characterization of the samples prepared by this method: (a) N_2 physisorption isotherms at 77 K for samples prepared at different times (shown in the legend of (c)) in a linear scale; (b) the corresponding NL-DFT pore size distributions obtained from the adsorption branch of the isotherms. (c) XRD diffractograms obtained for the same materials, graphite, marked as C (002) in the diffractograms, has been used as an internal standard for the calculation of the %MFI crystallinity. (d) Evolution of the micropore (green triangles) and mesopore volumes (dark blue rhombus) and the %MFI crystallinity (blue circles) with the time of treatment. (e) TEM micrograph of a sample prepared at 12 h of transformation. (f) TEM micrograph of the mesoporous region marked with a square symbol in (e).

3.7. Evolution of the Si/Al ratio of the obtained materials. Supplementary Figure 8 shows the evolution of the Si/Al ratio of the materials obtained as a function of the time. Their Si and Al content were calculated from the amount of Si and Al determined by ICP-OES/MS in the liquids of the reaction mixtures. The Si/Al ratios are very similar to the original CBV780, namely 40. It is well known that quaternary amines precipitate/avoid the dissolution of silica in basic environments, which helps to keep a high Si/Al ratio and excellent recovery yields.

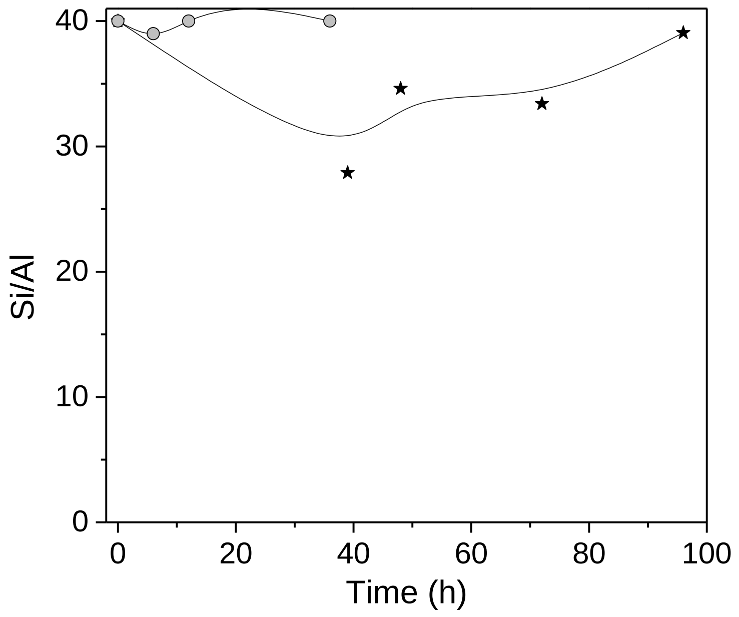

Supplementary Figure 8. Evolution of the Si/Al ratio with time for samples prepared using CTPABr (stars) or CTAB + TPABr (circles). Lines are interpolations to guide the eye.

3.8. Textural and structural characterization of physical mixtures of FAU and MFI zeolites. Physical mixtures of commercial FAU and MFI zeolites at different FAU:MFI weight ratios (2:1, 1:1 and 1:2).

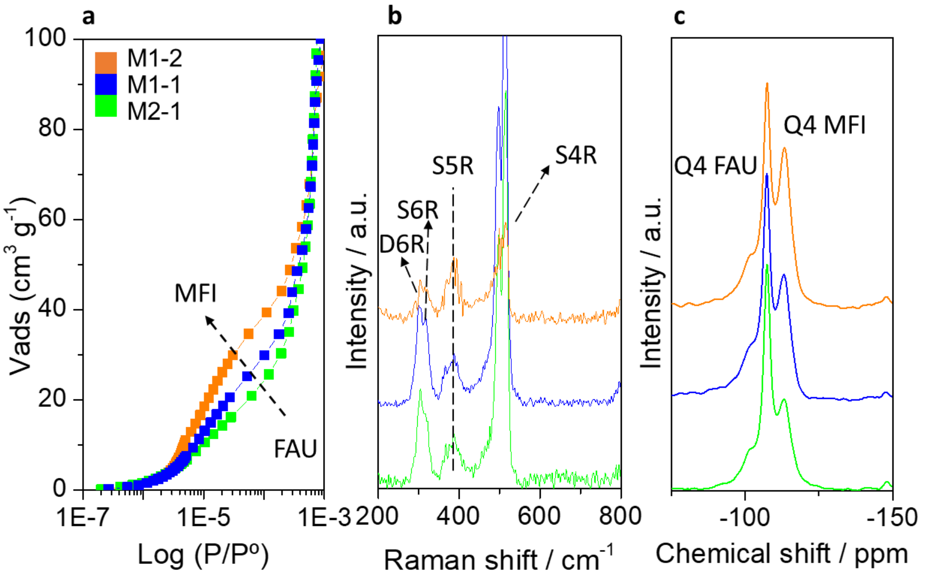

Supplementary Figure 9. Textural characterization of the physical mixtures of commercial FAU and MFI zeolites: (a) Ar physisorption isotherms at 77 K for samples prepared at different FAU:MFI weight ratios (shown in the legend of (b)) in a log scale; (b) UV-RAMAN spectra and (c) ^{29}Si NMR spectra for the same samples.

3.9. Field-Emission Scanning Electron Microscopy analysis of the samples:

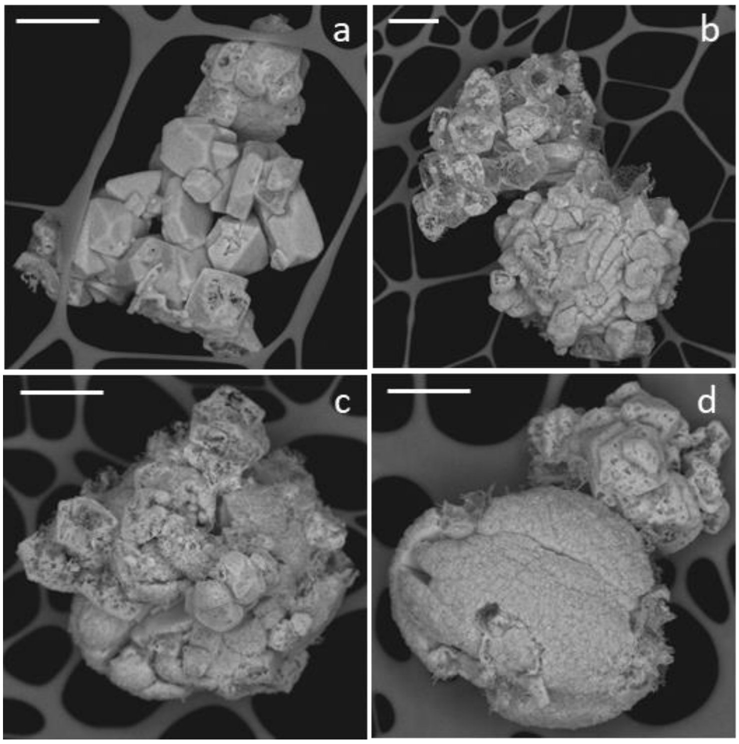

Supplementary Figure 10. FESEM images of samples prepared using CTPABr at different times of hydrothermal treatment, from left to right: A) 39, B) 48, C) 72 and D) 96 h. Scale bar corresponds to 1 μm.

3.10. Transmission Electron Microscopy analysis of selected regions:

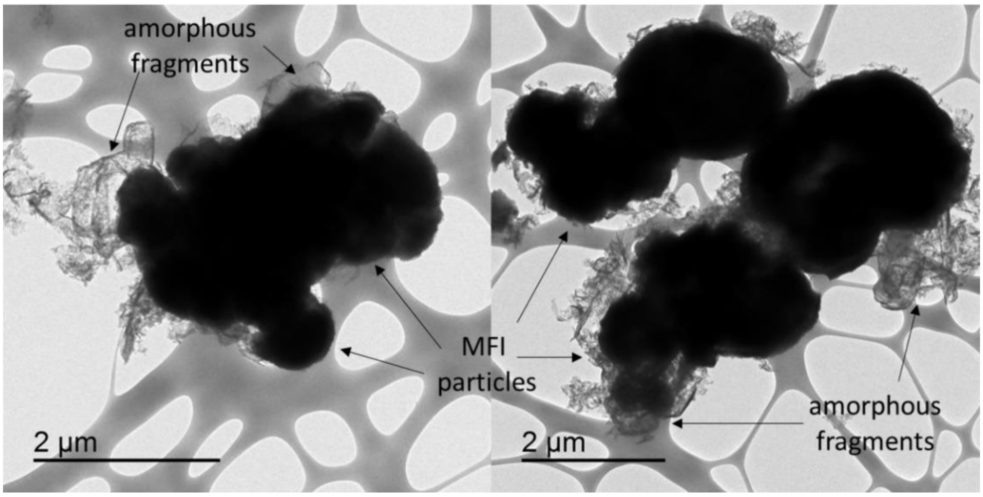

Supplementary Figure 11. TEM images of selected regions of sample prepared using CTPABr at 96 h, which suggest the assembly and attachment of fragments or particles from the amorphous phase to the new MFI phase.

3.11. Catalytic results of physical FAU:MFI mixtures and a mesoporous Al-MCM-41 material:

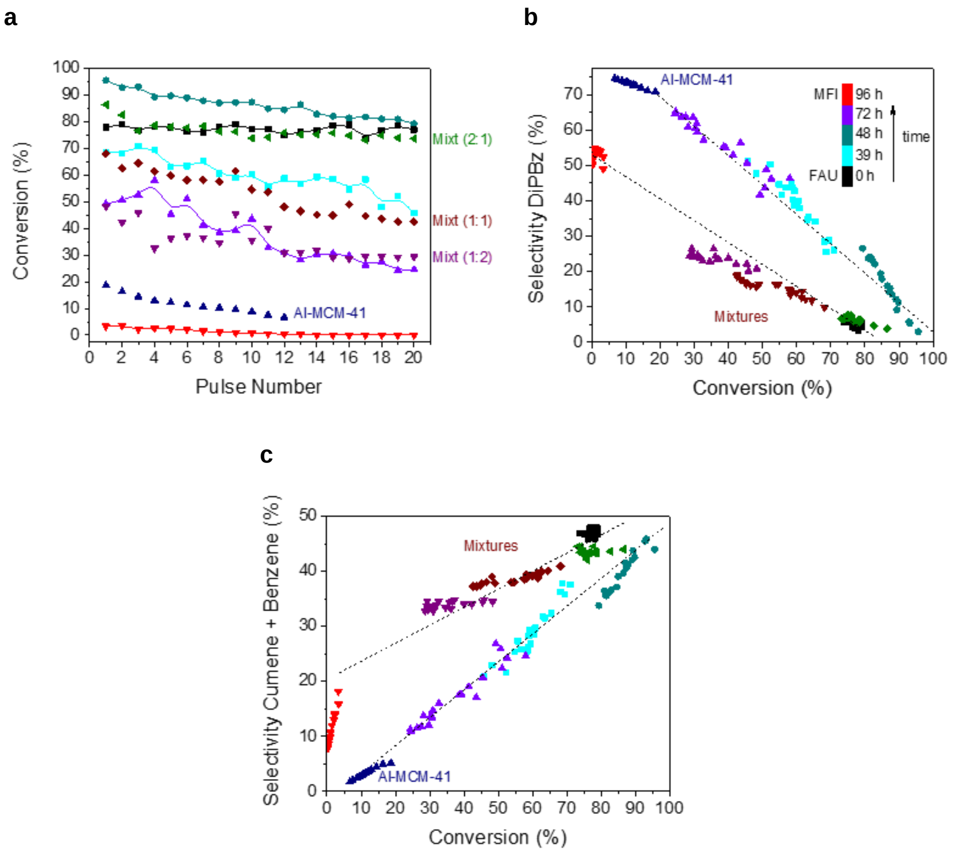

Supplementary Figure 12. Catalytic activity of the different materials evaluated showing the results of physical mixtures of FAU:MFI at different weight ratios (1:1, brown; 2:1, green; 1;2, purple), and an Al-MCM-41 type material (dark blue). (a) Performance of the materials in the catalytic cracking of TiPBz. (b) Evolution of the selectivity for 1,3-diisopropylbenzene with the increased conversion of TiPBz. (c) Evolution of the selectivity for cumene and benzene with the increased conversion of TiPBz.

3.12. ^{27}Al NMR analysis of selected materials:

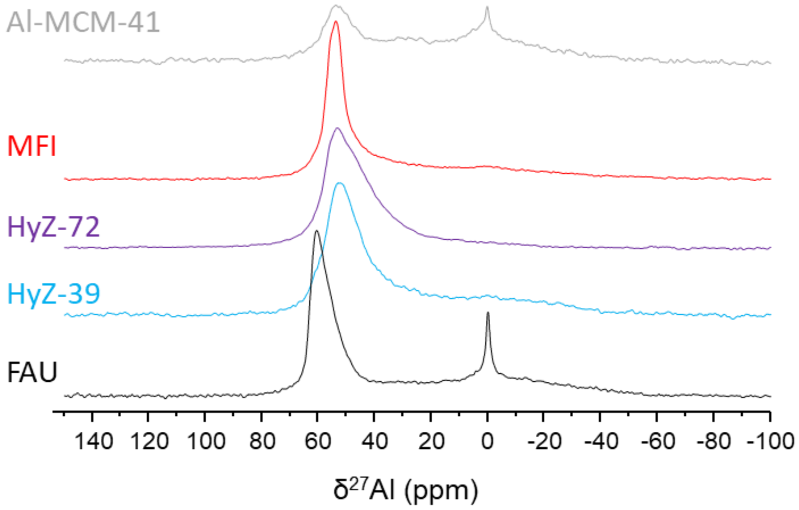

Supplementary Figure 13. ^{27}Al NMR spectra of the parent FAU, the intermediate samples treated at different times and the final MFI zeolite. The spectrum of an amorphous mesoporous Al-MCM-41 material has been included for comparison purposes.

Supplementary References

[1] A. Sachse, A. Grau-Atienza, E. O. Jardim, N. Linares, M. Thommes, J. García-Martínez, *Cryst. Growth Des.* **2017**, *17*, 4289–4305.

[2] M. J. Mendoza-Castro, E. Serrano, N. Linares, J. García-Martínez, *Adv. Mater. Interfaces* **2021**, *8*, 2001388.
